# An empirical investigation of college students’ acceptance of translation technologies

**DOI:** 10.1371/journal.pone.0297297

**Published:** 2024-02-26

**Authors:** Xiang Li, Zhaoyang Gao, Hong Liao

**Affiliations:** School of Foreign Languages and Cultures, Panzhihua University, Panzhihua, China; BRAC Business School, BRAC University, BANGLADESH

## Abstract

With the advancement of information technology and artificial intelligence, translation technologies have seen rapid development in language services and increasing integration in higher education. However, research on factors affecting students’ acceptance of these technologies remains limited. This study intends to formulate and test an extended Technology Acceptance Model (TAM) incorporating computer self-efficacy and perceived enjoyment to investigate students’ adoption of translation technologies. A questionnaire survey was conducted among 370 college students in China experienced with using translation technologies. The results from the structural equation modeling demonstrated a positive prediction on perceived ease of use and enjoyment from computer self-efficacy. Perceived enjoyment increased perceived ease of use and attitudes. Perceived ease of use positively influenced perceived usefulness and attitudes. Finally, attitudes positively predicted greater behavioral intentions to use translation technologies. However, computer self-efficacy was identified to have no significant effect on perceived usefulness. The study makes significant theoretical contributions by expanding TAM and offering practical guidance to improve students’ acceptance of translation technologies in tertiary education.

## Introduction

In the contemporary globalized era, the ability to communicate effectively across languages and cultures is of growing importance. The demand for translation services has seen a significant increase in recent years, leading to the development and widespread use of various translation technologies. As a result of the advancements in information technology and artificial intelligence, these technologies, which include machine translation tools, translation management systems, and computer-assisted translation software [1, 2], have the potential to facilitate the translation process, improve efficiency, and reduce errors [3, 4]. They also revolutionize the way people access and share information across linguistic barriers.

In a time when digital technologies pervade every aspect of our lives, the significance of translation technologies in the field of education has grown exponentially. These technologies not only facilitate language translation but also represent a bridge connecting diverse linguistic and cultural backgrounds within the global educational landscape. Teaching translation technologies in tertiary education has become a topic of growing interest among researchers, educators, and policymakers because these technologies play a crucial role in determining the academic achievements and career success of student translators. Despite their increasing importance in educational settings, the adoption of translation technologies among college students has not been thoroughly investigated. This knowledge gap warrants attention as recent statistics indicate the worldwide market for machine translation is projected to grow at a compound annual growth rate of 11.23% during 2023–2028, reaching an estimated value of 1.0424 billion USD by 2028, driven by the increasing trends of digitization [5]. Understanding the acceptance of translation technologies to revolutionize this burgeoning industry holds significant implications.

This research is theoretically grounded in the Technology Acceptance Model (TAM) [6], which is a commonly used framework that predicts user acceptance of emergent technologies on the basis of their perceived usefulness and perceived ease of use. While originally addressing general technology use, the TAM has been extended to explore the acceptance of various technologies across varying contexts [7–9]. It provides a robust foundation for understanding user attitudes and behaviors towards technologies, which makes it a suitable choice for the current study on translation technologies. In terms of translation studies, Yang & Wang [10] investigated factors affecting the intention to employ machine translation and the subsequent impacts of its usage based an extended TAM that included perceived ease of use, perceived usefulness, experience, motivation and behavioral intention. Recent studies have further identified additional variables that are essential in examining technology acceptance, specifically focusing on aspects like computer self-efficacy [11] and perceived enjoyment [12]. However, studies that examine the factors influencing the acceptance of translation technologies or integrate these factors to investigate their adoption, specifically within the context of higher education, remain sparse. Furthermore, factors encompassing technological, individual, and contextual dimensions, as well as their interaction, have yet to be comprehensively explored in affecting the intention to use translation technologies by college students.

As a result, this study aims to address the gap by conducting an in-depth examination of the acceptance of such technologies. The novelty of our approach lies in the integration of additional factors, specifically computer self-efficacy and perceived enjoyment, into the conventional TAM framework. This extension is critical, as it accounts for personal attributes and affective responses that significantly impact technology adoption, yet are often overlooked in traditional models. By investigating the factors that impact the acceptance of translation technologies among college students, our study provides crucial insights that can promote their effective use and maximize their potential benefits. This research is not only significant for the academic community, particularly those involved in language and translation studies, but also for developers of translation technologies and policymakers in the education sector. By understanding the factors that influence students’ acceptance of these technologies, we can better integrate them into educational practices, ultimately improving the quality of translation education and the preparedness of students for the professional world. In this context, the research will be guided by the following questions:

1. What are the key factors influencing college students’ acceptance of translation technologies?
2. How can the understanding of these factors and their interactions be applied to improve students’ acceptance of translation technology?
3. How might these findings be interpreted for translator training programs at the university level?

By answering these questions, this research offers a theoretical foundation for the examination of translation technology acceptance and broadens the applicability of TAM in the context of translation technologies, which provides an intricate understanding of how individual differences affect technology adoption in educational settings. The research is timely as it informs pedagogical and policy decisions during a transitional phase where student populations are encountering unprecedented exposure to specialized language technologies. By supplying evidence-based recommendations, the results will provide insights into how these technologies can be effectively integrated into the educational practices, thereby significantly contributing to the development of future translation professionals equipped for the modern, technology-driven world.

## Literature review and hypothesis

### Theoretical background

The TAM proposed by Davis in 1989 is an adaptation of the Theory of Reasoned Action (TRA) originally developed by Fishbein and Ajzen [13]. The TRA is a widely accepted model that predicts and explains human behavior in specific contexts. It proposes that individual behavior is primarily influenced by behavioral intentions, which are subsequently a function of an individual’s attitude towards the behavior itself and subjective norms associated with the performance of the behavior [13]. Building on the TRA, Davis [6] proposed the TAM to elucidate computer-usage behavior. The TAM suggests that an individual’s behavioral intention to use a technology is influenced by their perceived usefulness and perceived ease of use of the technology. Perceived usefulness can be characterized as an individual’s belief in the potential of a specific system to enhance job performance, while perceived ease of use pertains to the individual’s conviction about the effortless operation of the system [6].

The TAM has found extensive application across various fields to specifically assess user acceptance and adoption of new information systems and technologies [14, 15]. However, it has been subjected to various adaptations and extensions to incorporate additional variables and constructs over the years [16, 17]. For instance, Venkatesh and Davis [18] introduced an extended version of the TAM, referred to as TAM2, that incorporated subjective norms, image, job relevance, output quality, result demonstrability, voluntariness and experience into the original model. Later, Venkatesh et al. [19] proposed the Unified Theory of Acceptance and Use of Technology (UTAUT), which integrated eight distinct models, including the TAM, offering a comprehensive framework to explain technology usage intentions and behaviors.

In educational contexts, TAM has been widely employed to assess the acceptance of various digital tools and learning technologies. Its robust framework aligns well with the dynamics of technology use in educational environments, where both ease of use and perceived utility significantly influence students’ technology adoption decisions. By focusing on these two core TAM constructs, we can gain deeper insights into the specific factors that drive the acceptance of translation technologies among college students. While TAM’s original constructs of perceived ease of use and perceived usefulness provide a solid foundation, recent studies suggest that additional factors can further enrich our understanding of technology acceptance behaviors in higher education. In this study, we extend TAM by incorporating computer self-efficacy and perceived enjoyment. Computer self-efficacy reflects an individual’s belief in their ability to use computers effectively [20, 21], which can significantly impact their perception of a technology’s ease of use and usefulness. Perceived enjoyment, the extent to which using technology is enjoyable in its own right [22, 23], is increasingly recognized as a crucial factor in the technology adoption process, particularly among younger users in educational settings.

Translation technologies, characterized by their unique blend of linguistic and technical challenges, make the TAM framework particularly apt for the present study. The perceived ease of use is vital in understanding how students perceive the complexity and user-friendliness of these technologies, while perceived usefulness reflects the technologies’ perceived effectiveness in aiding their educational pursuits. By extending TAM with computer self-efficacy and perceived enjoyment, we aim to capture a more intricate understanding of the factors influencing students’ acceptance of these technologies. This extended model acknowledges the role of individual self-efficacy in managing technology and the intrinsic motivation derived from enjoyable technological interactions. This choice is also motivated by the robustness of the TAM in establishing a foundation to track the influence of external factors on internal attitudes and intentions [23]. Furthermore, the conciseness of the model have proven conducive to adapt its application across diverse populations and technology-related contexts [11, 24]. As a result, the inclusion of these external aspects in technology acceptance would significantly enhance and broaden the explanatory capacity of TAM, making it even more relevant and applicable to the context of translation technologies in higher education.

### Research hypothesis

#### Computer self-efficacy

The concept of computer self-efficacy is rooted in the broader notion of self-efficacy, a key component of the Social Cognitive Theory. Self-efficacy elaborates an individual’s self-belief in their own capabilities, which in turn can shape one’s actions, persistence, efforts, and determination during challenging circumstances [25]. Computer self-efficacy specifically pertains to an individual’s assessment of their capacity to manipulate a computer [20, 21] and one’s belief in their ability to easily complete particular tasks by leveraging a computer [16, 26]. Based on the Social Cognitive Theory, individuals lacking self-confidence in their capabilities might not deploy the requisite effort to accomplish a task, or show persistence in overcoming potential obstacles. Therefore, computer self-efficacy is not a marker of an individual’s computer skills per se, but it sheds light on what they anticipate they would be capable of doing with their computer skills in the future [27].

New technologies are accepted and utilized more readily when people are self-efficacious with their computers [28]. The degree of self-efficacy influences his/her willingness and ability to gather cognitive resources, motivation, and develop an action plan to address situational demands. It is also suggestive of the determination, goals, and effort the individual will deploy to complete tasks and surmount challenges [29]. Additionally, individuals exhibiting lower computer self-efficacy often encounter barriers in performance, consequently adjusting their perception of their technical skills negatively. On the contrary, those possessing higher computer self-efficacy demonstrate resilience towards intricate challenges, persistently striving to overcome difficulties, thereby fostering an increased propensity to utilize technology [30, 31].

As for college students’ acceptance of translation technologies, the role of computer self-efficacy becomes even more significant. Translation technologies, which often involve complex interfaces and functions, require a certain level of computer literacy and confidence. As such, students’ belief in their capability to use these technologies effectively can profoundly impact their acceptance and utilization of these tools.

#### Computer self-efficacy and perceived usefulness

Computer self-efficacy has been widely recognized as a contributing factor to the acceptance of technology [30]. This is supported by a multitude of empirical studies that have established a strong correlation between computer self-efficacy and the perceived usefulness of various technologies [17, 14]. For instance, Jiang et al. [32] showed that university students with high computer self-efficacy tend to appreciate the value and benefits of online learning platforms, because they could feel more confident in operating these platforms, which enhances their perceived usefulness. Similarly, Al-Rahmi et al. [33] argued that students who perceive themselves as capable of using computers are more likely to leverage Information and Communication Technologies (ICTs) that allow them to be more productive, thereby increasing their perceived usefulness. This is also echoed by Mensah et al. [11], who found that computer self-efficacy significantly affected the perceived usefulness of an e-learning system among college students. Building on this foundational knowledge and given the accelerating growth of translation technologies, it becomes imperative to understand the role of computer self-efficacy in this context. Especially since translation technologies, like any other advanced tools, require a certain level of confidence in using computer-based resources. Hence, this study put forward the following hypothesis.

Hypothesis 1: Computer self-efficacy positively impacts perceived usefulness of translation technologies.

#### Computer self-efficacy and perceived ease of use

The consistent positive influence of computer self-efficacy on the perceived ease of use across various technologies has been well-documented in academic research. For example, Teo et al. [34] found that teachers with high computer self-efficacy tend to perceive technology as easy to use. Similarly, a study on software testers identified computer self-efficacy as a robust predictor of perceived ease of use of software testing tools [35]. The correlation has also been found in the context of e-learning. Thongsri et al. [36] confirmed that Chinese students with high computer self-efficacy perceived e-learning as easier to use and more beneficial for achieving academic goals. This was echoed by Salloum et al. [30], who found that students in the United Arab Emirates with high computer self-efficacy perceived e-learning systems as more user-friendly. In the context of translation technologies, it may be hypothesized that individuals possessing high computer self-efficacy will deem these technologies as relatively simple to operate because they are more inclined to surmount technological challenges and less likely to feel frustrated when using these technologies [37]. Based on the above arguments, this study hypothesized the following statement.

Hypothesis 2: Computer self-efficacy positively impacts perceived ease of use of translation technologies.

#### Computer self-efficacy and perceived enjoyment

Perceived enjoyment originates from an intrinsic belief shaped by an individual’s subjective interaction with a system, which measures the degree to which the usage of a particular system is considered enjoyable, exclusive of any resulting performance outcomes associated with the system’s use [22, 23]. Existing studies conducted on undergraduate students in Romania and Spain, and students from two Saudi universities revealed notable impacts of self-efficacy in computer use on perceived enjoyment towards using web-based technologies and digital learning respectively [15, 38]. Moreover, it has been found that students demonstrating a high degree of computer self-efficacy are more probable to express confidence in blogging, which in turn elevates the perceived enjoyment derived from the blogging experience [39].Therefore, it can be hypothesized that computer self-efficacy might have a positive effect on perceived enjoyment towards using translation technologies.

Hypothesis 3: Computer self-efficacy positively impacts perceived enjoyment towards using translation technologies.

#### Perceived enjoyment and perceived ease of use

Perceived enjoyment has significantly shown its impact on the perceived ease of using technology across various research. For example, perceived enjoyment influenced college students’ perceived ease of using the Internet for learning [40]. This finding was further supported by Abdullah and Ward [41] who found that perceived enjoyment significantly influenced perceived ease of use in e-learning acceptance among students in the UK. In this regard, students are likely to perceive an e-learning system as user-friendly if they find it enjoyable [30]. In the meanwhile, Park et al. [42] proposed that the enjoyment derived from the utilization of information science reduces cognitive load, potentially leading to an underestimation of the task difficulty and consequently enhancing user perceptions of its ease of use. Consequently, the study formulated the following hypothesis.

Hypothesis 4: Perceived enjoyment positively impacts perceived ease of use of translation technologies.

#### Perceived enjoyment and attitudes towards use

Several studies also suggested that perceived enjoyment and attitudes towards the use of diverse technologies are significantly correlated [43]. Users are more likely to adopt positive attitudes toward technologies if they find interaction with them enjoyable. For instance, research on AI-enabled fitness apps suggests that the perceived enjoyment derived from using these apps positively influences individuals’ attitudes towards them [44]. With regard to education field, an investigation encompassing 317 graduates and undergraduates showed that the perceived enjoyment positively influenced the attitudes towards the application of ICTs in higher education learning environments in Saudi Arabia [45]. Another study, which sampled 360 college students in Ghana, found that students’ perspectives on the utilization of social media platforms for collaborative learning were shaped significantly by their perceptions of enjoyment [12]. Taking these studies into account, it is reasonable to put forward the following hypothesis.

Hypothesis 5: Perceived enjoyment positively impacts attitudes towards using translation technologies.

#### Perceived ease of use and perceived usefulness

Perceived ease of use significantly influences the perceived usefulness of various technologies because individuals are inclined to perceive a technology as useful if they view it as straightforward to learn and operate [46]. On the contrary, if a technology is perceived as complex or challenging to apply, potential users are apt to seek other alternatives or resort to familiar formats, regardless of its perceived usefulness [47]. This is particularly relevant in an educational context, when students perceive the application of technology as requiring minimal effort, they are more inclined to integrate it into their learning routines [40]. Such assumption is supported by studies conducted in Turkey, China, Saudi Arabia, and Jordan, where perceived ease of use significantly influenced students’ perceptions of usefulness in terms of learning management systems, the Internet, distance education platforms, online learning system, and metaverse technology for education [9, 48–51]. Therefore, we hypothesized that making translation technologies easy to use could increase their perceived usefulness among college students.

Hypothesis 6: Perceived ease of use positively impacts perceived usefulness of translation technologies.

#### Perceived ease of use and attitudes towards use

The TAM indicates that perceived ease of use significantly influences users’ attitudes towards technology [52], which is supported by a variety of studies across different contexts. For instance, research on academicians in Saudi Arabia found that perceived ease of use was the most influential factor in shaping their attitudes towards e-learning [53]. Similarly, a study on Chinese college students found a positive relationship between perceived ease of use and attitudes towards online learning [54]. Further evidence found that perceived ease of use of digital tools positively impacted attitudes towards their use in higher education [55]. Nevertheless, it is noteworthy that some studies have found no significant effect of perceived ease of use on attitudes [56, 57]. These contrasting findings suggest that while perceived ease of use generally yields a positive impact on attitudes relating to technology utilization, this relationship may be influenced by other factors or vary in different contexts. Therefore, Hypothesis 7 was proposed to test whether perceived ease of use significantly influences students’ attitudes towards using translation technologies.

Hypothesis 7: Perceived ease of use positively impacts attitudes towards use translation technologies.

#### Perceived usefulness and attitudes towards use

The TAM also indicates that the perceived usefulness of a system can influence users’ attitudes towards it [6]. When users believe that the utilization of a specific information system could bolster their professional or academic performance [46], they would develop a positive attitude towards using that technology [31]. This relationship was also verified in the context of digital technologies in tertiary education [55]. For instance, the attitude towards the application of ICT-integrated mathematics instruction was positively impacted by the perceived usefulness [58]. Similarly, perceived usefulness was observed to maintain a positive correlation with the attitudes of students towards e-learning in colleges and universities in Saudi Arabia [59]. Based on these arguments, the perception of a technology’s usefulness is a crucial factor influencing users’ attitudes towards it. Therefore, we hypothesized the following argument.

Hypothesis 8: Perceived usefulness positively impacts attitudes towards the use of translation technologies.

#### Attitudes towards use and behavioral intention to use

Attitude, as defined by Ajzen and Fishbein [60], is a person’s affective evaluation towards a specific task. In the context of technology use, it refers to the degree to which users either appreciate or despise the utilization of a technology. This attitude influences an individual’s behavior through the processing of information and the formation of perceptions regarding their environment [53]. The TAM revealed that the behavior intentions are influenced by individuals’ attitudes towards technology utilization [6]. In terms of educational settings, several studies have found that students’ attitude serves as a crucial predictor for explaining their intent to use e-learning systems [61] and that positive attitudes towards using computers affected students’ intentions to use ICT [33]. In the context of translation technologies, it can be inferred that learners with a positive attitude will likely have a continued willingness to use these technologies. Hence, we proposed the following hypothesis.

Hypothesis 9: Attitudes towards use positively impacts behavioral intention to use translation technologies.

**Fig 1:**
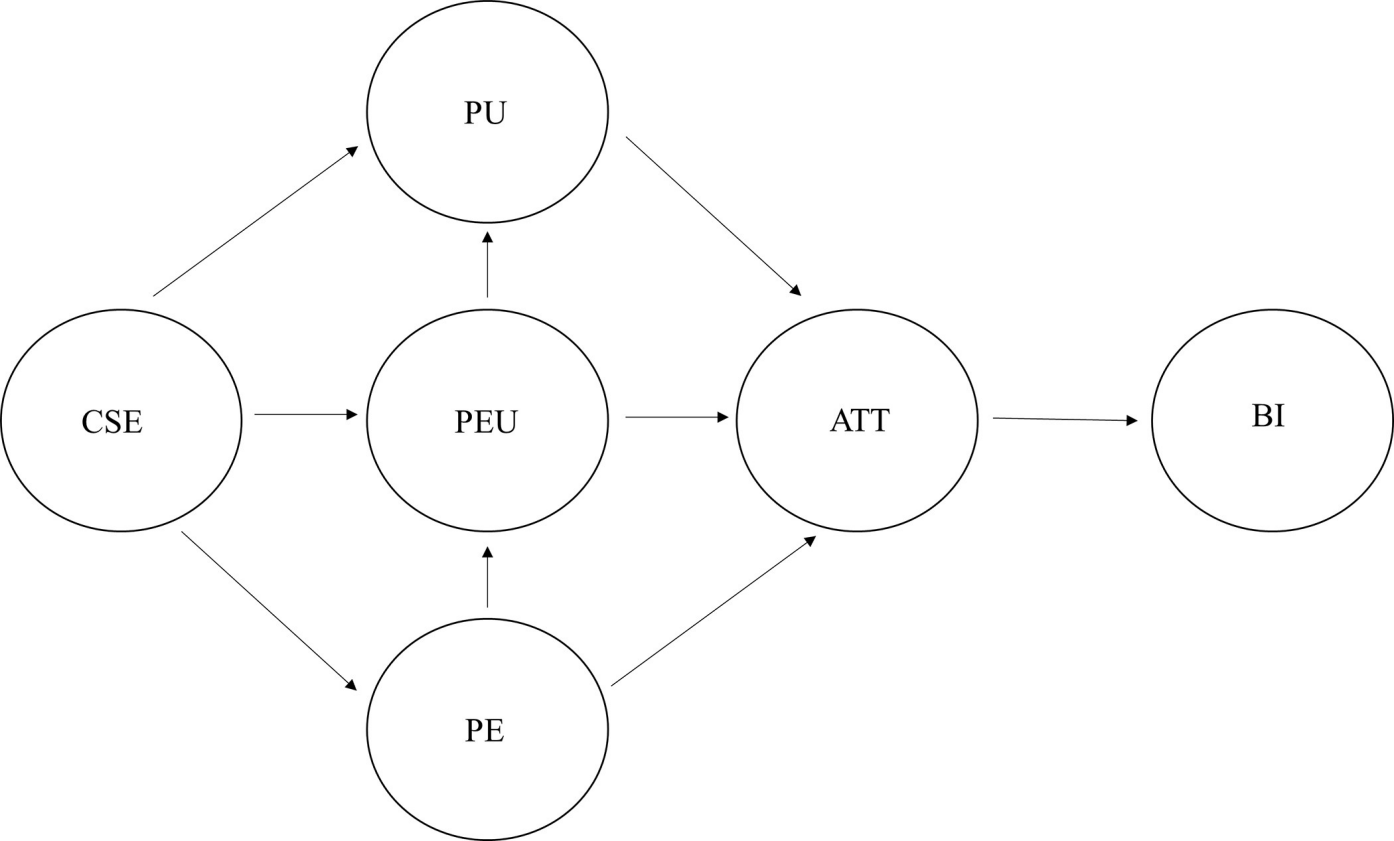
Research model. Note: CSE = Computer self-efficacy; PE = Perceived enjoyment; PU = Perceived usefulness; PEU = Perceived ease of use; ATT = Attitude toward use; BI = Behavioral intention.

The research model proposed in this study is presented in Fig 1.

## Material and methods

### Participants

The target population for this study was upperclassmen college students in China who have experience with using translation technologies. More specifically, the accessible population included juniors and seniors majoring in translation and interpretation enrolled at a public university in China. These students were selected as a case study because they represent a group that frequently interacts with translation technologies in their academic pursuits. These technologies are often integrated into the curriculum of translation training courses within most Chinese universities during their third and fourth academic years. Such practice was also observed in the university where this investigation was conducted. This demographic is particularly relevant for our study as it provides insights into the attitudes and acceptance of translation technologies among young adults who are native Chinese speakers and are learning English as a second language.

The data was collected using an online questionnaire survey that allowed for a wide distribution and facilitated the collection of responses. A convenience sampling technique was adopted to recruit participants. Convenience sampling is an appropriate non-probability sampling method for researchers who need to recruit participants that meet specific criteria and are conveniently available to participate [62]. This approach is widely used in exploratory research where the focus is on obtaining insights quickly and economically, especially when working with a population that is easily accessible [63]. Furthermore, previous technology acceptance studies have effectively utilized convenience sampling [27, 49].

370 students voluntarily participated in the study from June 29, 2023 to July 16, 2023. This concurs with the recommended guidelines for ascertaining an adequate sample size in structural equation modeling. As per the widely accepted rule of thumb, it is advisable to have 10 cases per variable [64]. Considering there were 20 observable variables involved in this study, a sample comprising 200 participants is regarded as sufficient. The voluntary nature of participation ensured that the responses were genuine and reflected the students’ true attitudes and experiences with translation technologies. In addition, the objectives of the research were clearly communicated to the students before they completed the questionnaire. They were informed that their responses would be anonymized and used solely for the purpose of this study. Their consent to participate was obtained in a written electronic form after they were provided with the instruction. This ensured that the students were aware of their rights as participants and that the study adhered to ethical research practices. The School of Foreign Languages and Cultures’ Ethics Committee at Panzhihua University granted ethical approval for this research in accordance with the Declaration of Helsinki (Approval No. HRECA23-001). This approval ensured the study’s adherence to both local and international guidelines for human research.

### Instruments

The study utilized a structured questionnaire as an instrument, designed on the foundations of previously validated literature. There were two parts to the questionnaire. Part One collected demographic information, which was self-reported by the participants and included details such as age, gender, grade, and family residence. This information was collected to provide a comprehensive profile of the participants.

The second part of the questionnaire was composed of six subscales, each measuring a different construct related to the acceptance of translation technologies. Each of these constructs was measured using several items answered on a 5-point Likert scale, ranging from “strongly disagree” to “strongly agree”. The items for each construct were derived from various sources. To be specific, four items measuring Computer Self-Efficacy were adapted from Hsu et al. [65]. Perceived Enjoyment was measured using three items adapted from Gurban & Almogren [59]. Perceived Usefulness, Perceived Ease of Use, and Behavioral Intention to Use were measured with three items adapted from Davis [6] respectively. Attitude Toward Use was measured by four items adapted from Teo & Zhou [31]. These items have been demonstrated to be statistically reliable in previous studies, thus ensuring the validity and reliability of the instrument utilized in this research.

To ensure the accuracy and appropriateness of the questionnaire for our target participants, a meticulous two-step validation process was undertaken. Firstly, the questionnaire was translated and reviewed by three professional translators, all possessing national accreditations for translators. Secondly, a pre-test was conducted with 7 randomly selected students who were acquainted with translation technologies. Their feedback led to the rephrasing of two items in Chinese. Such meticulous attention to detail not only ensured the linguistic precision but also amplified the content validity of the instrument.

### Data analysis

First, we performed a reliability analysis of the data. This was done using SPSS 27.0, a tool that is widely used in social science research for its ability to handle large datasets and perform a variety of statistical tests. The reliability analysis was performed to determine that the survey instrument used in the study was consistent and reliable. This involved calculating Cronbach’s alpha for each of the constructs in the research. SPSS was also employed for the performance of descriptive analysis which was instrumental in understanding the basic characteristics of the data in the study. Following this, Confirmatory Factor Analysis (CFA) and Structural Equation Modeling (SEM) were conducted using Amos 24.0 to provide evidence of construct, convergent and discriminant validity and test the proposed hypothesis.

## Results

### Demographic description

As shown in Table 1, there were 370 students involved in this investigation. 316 were female students, accounting for most of the proportion with 85.4%. Male counterparts totaled 54, representing a smaller portion, 14.6% of the total sample. In terms of grade, juniors represented the majority with 212 respondents, amounting to 57.3% of the total participants. Seniors, who made up 158 of the respondents, corresponded to the remaining 42.7%. As for the age distribution among the respondents, the vast majority were between 18 and 22. They totaled 351 students, which accounted for 94.9% of the total. One respondent was younger than 18 (making up for about 0.3% of the total number), and 18 respondents (or approximately 4.9%) were aged above 22. Regarding the family socio-economic background of the students involved in the study, most respondents came from rural areas. Those from a rural background (250 respondents) accounted for as much as 67.6% of the total sample. Participants from urban families were significantly fewer, totaling only to 120 participants or 32.4% of the total.

**Table 1:** Demographic description of the participants.

Demographics	Category	Frequency	Percentage (%)
Gender	Male	54	14.6
	Female	316	85.4
Grade	Junior	212	57.3
	Senior	158	42.7
Age	<18	1	0.3
	18–22	351	94.9
	>22	18	4.9
Family Residence	Urban	120	32.4
	Rural	250	67.6

### Common method deviation and multicollinearity test

To gauge the presence and extent of common method bias in our study, we employed the Harman one-factor test, a commonly acknowledged approach for evaluating common method bias [66]. In our research, the result of the principal components analysis showed that the first factor accounted for 28.277% of the total variance, which meets the typically accepted threshold of 50% [67]. Consequently, it is reasonable to infer that the common method bias is not present to a significant degree within our research.

To assess multicollinearity among the predictor variables in our analysis, we employed the Variance Inflation Factor (VIF) as the diagnostic tool. Utilizing SPSS 27.0, we computed VIF values for each of the five independent variables, namely, computer self-efficacy, perceived enjoyment, perceived ease of use, perceived usefulness, and attitude toward use, by designating behavioral intention as the dependent variable. The resultant VIF values were 1.434, 2.787, 2.092, 2.429, and 3.088, respectively. As none of these values exceeded the conventional threshold of 5.0 [68], it can be confirmed that multicollinearity does not pose a concern for the current study.

### Measurement model

CFA was performed to validate the measurement model, which comprises six latent constructs. To evaluate these constructs, an examination of their reliability, as well as their convergent and discriminant validities was conducted. The reliability of the questionnaire was evaluated via Cronbach’s Alpha, which showed values exceeding 0.90 for all constructs, as presented in Table 2. This surpasses the recommended threshold of 0.7 [69], thus indicating high internal consistency of all constructs. Notably, all item factor loadings showcased values ranging from 0.789 to 0.956, providing further support for the model’s reliability. Furthermore, the constructs displayed a Composite Reliability (CR) exceeding 0.90, well above the standard cut-off of 0.70 [68]. In terms of the Average Variance Extracted (AVE), all constructs surpassed the recommended level of 0.50 [70], with values all above 0.70, showcasing good convergent validity within the model. For discriminant validity, an examination was carried out following Fornell & Larcker’s [70] recommendation, where the square root value of each construct’s AVE should surpass its correlation coefficients with all other constructs. Table 3 shows that this study’s findings align with this requirement, with the square root of all AVEs exceeding their corresponding correlation coefficients, thereby ensuring the constructs’ discriminant validity.

**Table 2:** Construct validity and convergent validity.

Construct	Item	Factor Loading	CR	AVE	Cronbach’s Alpha
CSE	CSE1	0.836	0.916	0.733	0.913
	CSE2	0.946			
	CSE3	0.845			
	CSE4	0.789			
PE	PE1	0.806	0.908	0.767	0.905
	PE2	0.896			
	PE3	0.921			
PU	PU1	0.938	0.943	0.847	0.942
	PU2	0.88			
	PU3	0.942			
PEU	PEU1	0.885	0.907	0.766	0.904
	PEU2	0.932			
	PEU3	0.804			
ATT	ATT1	0.863	0.924	0.754	0.921
	ATT2	0.859			
	ATT3	0.814			
	ATT4	0.933			
BI	BI1	0.822	0.920	0.793	0.911
	BI2	0.956			
	BI3	0.889			

Note: CSE = Computer self-efficacy; PE = Perceived enjoyment; PU = Perceived usefulness; PEU = Perceived ease of use; ATT = Attitude toward use; BI = Behavioral intention; CR = composite reliability; AVE = average variance extracted.

**Table 3:** Discriminant validity.

Items	CSE	PE	PU	PEU	ATT	BI
CSE	0.856					
PE	0.549***	0.876				
PU	0.398***	0.663***	0.92			
PEU	0.474***	0.643***	0.738***	0.875		
ATT	0.444***	0.676***	0.752***	0.816***	0.868	
BI	0.436***	0.576***	0.593***	0.641***	0.763***	0.891

### Structural model

The analysis of the structural model was carried out by using the constructs that were established earlier. The aim was to better understand the relationships between each construct, specifically their effects and interactions. The statistical fitness of this model was measured through a number of indices [71], with their associated thresholds shown in Table 4, which verifies the data’s adequacy and the model’s validity.

**Table 4:** Model fit index.

Fit Index	*χ* ^2^	*df*	*χ*^2^/*df*	RMSEA	SRMR	GFI	NFI	TLI	CFI
Threshold	-	-	<5	<0.05	<0.05	>0.90	>0.90	>0.90	>0.90
Model	339.444	155	2.19	0.057	0.0379	0.918	0.953	0.968	0.974

**Fig 2:**
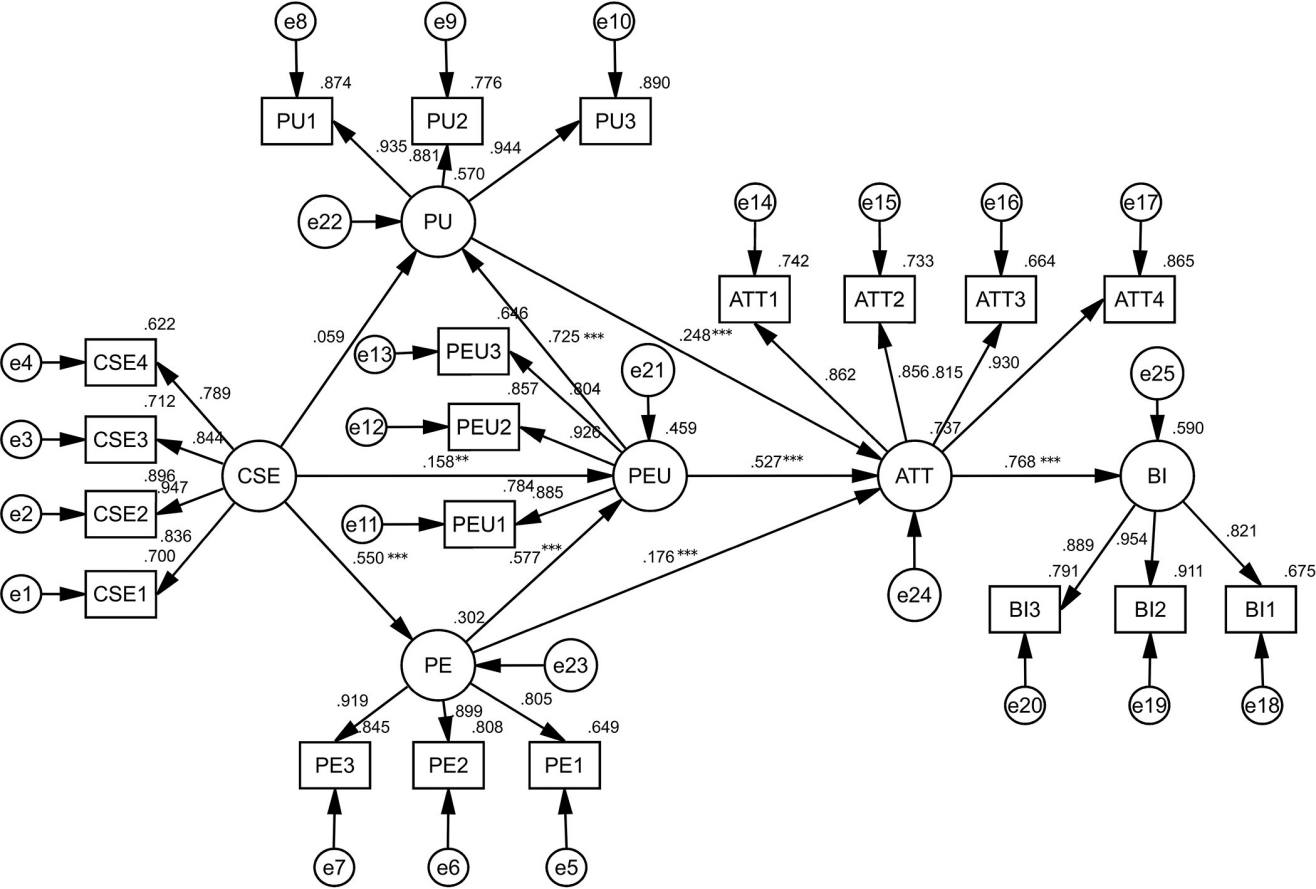
Result of the path analysis. *** p < 0.001, ** p < 0.01.

The empirical findings, displayed in Fig 2 and Table 5, highlighted the model’s efficiency, accounting for 59% of the variance in the behavioral intention to use translation technology among college students. The data in Table 5 further illuminated that computer self-efficacy positively predicted perceived ease of use (β = 0.158, p < 0.01) and perceived enjoyment (β = 0.55, p < 0.001), implying that students with higher confidence in computer skills found translation technologies easy and enjoyable to use. In addition, perceived enjoyment had a positive and significant impact on the perceived ease of use (β = 0.577, p < 0.001), suggesting that the greater the enjoyment perceived by the user, the easier they find the technology to use. Further, perceived ease of use was found to be a significant predictor of perceived usefulness (β = 0.725, p < 0.001), indicating that if the technology was found to be user-friendly, it was also considered useful. It also highlighted that perceived enjoyment (β = 0.176, p < 0.001), perceived usefulness (β = 0.248, p < 0.001), and perceived ease of use (β = 0.527, p < 0.001) showed significant positive influences on the attitude toward using translation technologies. Positive attitudes towards use subsequently led to a significantly higher behavioral intention to use translation technologies (β = 0.768, p < 0.001). Nevertheless, no significant connection was found between computer self-efficacy and perceived usefulness (β = 0.059, p >0.05), implying that inherent computer skills do not necessarily lead to an individual finding translation technology useful.

**Table 5:** Hypothesis testing.

Hypothesis	Path	Standardized Coefficient	S.E.	C.R.	P-value	Test Results
H1	PU←CSE	0.059	0.042	1.285	0.199	Rejected
H2	PEU←CSE	0.158	0.046	2.923	**	Supported
H3	PE←CSE	0.550	0.051	9.914	***	Supported
H4	PEU←PE	0.577	0.055	9.624	***	Supported
H5	ATT←PE	0.176	0.043	3.757	***	Supported
H6	PU←PEU	0.725	0.055	14.25	***	Supported
H7	ATT←PEU	0.527	0.066	7.861	***	Supported
H8	ATT←PU	0.248	0.049	4.628	***	Supported
H9	BI←ATT	0.768	0.051	14.724	***	Supported

*** p < 0.001, ** p < 0.01

## Discussion

The objective of this study was to investigate the factors influencing college students’ acceptance of translation technologies by proposing and testing an extended TAM. Consistent with the TAM theory, the results demonstrate that perceived usefulness, perceived ease of use and attitude toward use contribute to the acceptance of translation technologies among students. Notably, our findings expand the TAM theory by highlighting the importance of other pivotal factors which specifically include computer self-efficacy and perceived enjoyment on students’ intention to use translation technologies.

As corroborated by the findings, computer self-efficacy was identified to positively impact perceived enjoyment and perceived ease of using translation technologies. This aligns with previous studies showing that higher computer self-efficacy leads to greater perceived ease of use and enjoyment of various technologies [30, 34, 36]. The results of this research reinforce the idea that computer self-efficacy, as encapsulated by students’ confidence in learning a variety of computer skills, the perception that computers are easy to use, their ability to operate a computer skillfully, and the ease of achieving desired outcomes using a computer, has a substantial bearing on students’ perception and acceptance of translation technologies. The items of computer self-efficacy in our study reflect a broader scope of computer skills rather than just the mere operation of software, hinting at a holistic relationship between confidence in using technology and the acceptance of specialized tools like translation technologies. Furthermore, perceived enjoyment, stemming from the pleasure students derive from learning and using translation technology and the fun they associate with it, is directly influenced by their computer self-efficacy. This dynamic is evident when considering that students who feel adept at using computers are more likely to find the learning process enjoyable. Their self-belief in managing technology effectively translates to a positive emotional experience with translation technologies. Similarly, perceived ease of use, determined by the clarity of interactions with translation technology, the facility to command the technology, and its overall user-friendliness, is strengthened by strong computer self-efficacy. When students perceive themselves as competent in computer skills, they would find interacting with translation technologies “clear and understandable” [6] and easy to manipulate for their requirements. They could also derive more enjoyment from the process, viewing interactions with technology as rewarding challenges rather than frustrating barriers. Since computer self-efficacy embodies an individual’s belief in their capacity to apply their skills proficiently in the future [21], students with elevated levels of self-efficacy tend to exhibit enhanced motivation and persistent in overcoming difficulties when using translation technologies, viewing them not as a cumbersome tool but rather as an enhancer of their skills and capabilities.

However, no significant correlation was identified between computer self-efficacy and perceived usefulness, contrasting some earlier studies [32, 33]. A potential explanation is that while the items related to computer self-efficacy address a general confidence in using computers, they do not pinpoint a specific proficiency in using translation technologies. As suggested by Al-Adwan [27], computer self-efficacy evaluates individuals’ self-belief in computer application, which is distinct from their tangible computer skills. Therefore, while students might be confident and competent in broader computer use, this does not inherently suggest they perceive translation technologies as enhancing their translation performance or productivity. The usefulness likely depends more on how the technology fits their translation needs rather than their general computer abilities. Another potential explanation is that translation technologies require specialized skills beyond basic computer literacy. Thus, general computer self-efficacy may not translate to perceiving translation tools as more useful.

Perceived enjoyment was observed to significantly influence perceived ease of use and attitudes towards the use of translation technologies among college students. This reinforces the proposition that technology perceived as enjoyable influences users’ perception of its ease of use [30]. In the context of translation technologies, students who experience fun and enjoyment while using these tools tend to overlook potential difficulties and develop more favorable attitudes. A noteworthy mechanism that has emerged from our research, and which has been similarly identified by Park et al. [42], is the potential of perceived enjoyment to diminish cognitive burden. In essence, when users find the technology pleasurable, they tend to perceive it as less mentally taxing, leading to an effortless engagement. This phenomenon underscores why students may find enjoyable technologies less daunting and more approachable. Specifically, if students find the process of using translation technology pleasant, they might perceive their interactions with the technology as clearer, more manageable, and straightforward. Moreover, the items designed to measure perceived enjoyment in this study emphasize the importance of ensuring that the process of learning and implementing the technology is perceived as pleasant and captivating. This is consistent the broader perspective that for technology adoption to be successful, it should not just be functional but also cater to the emotional and experiential aspects of the user [72]. In brief, while the intrinsic nature of translation technologies revolves around facilitating the process of translation, a layer of enjoyment embedded within their design and functionality can significantly boost their acceptance among the student population.

The findings from this research reaffirm the integral connection between perceived ease of use and the subsequent perceived usefulness of translation technologies among college students. As predicted and aligned with the TAM, a technology that is perceived to be user-friendly and simple to interact with is also likely to be perceived as more beneficial by its users. This phenomenon is consistent with past literature, as seen in the experiences of students across multiple nations with different learning management systems, online platforms, and digital tools [48, 49, 51]. Specifically, when students felt that their interaction with translation technologies was clear and intuitive, they were more likely to believe that the technologies would boost the quality and productivity of their translations. Likewise, the ease of use was positively linked to students’ enthusiasm and enjoyment in using the technologies, with many finding them both fun and an exciting addition to their academic tasks. Furthermore, the clear connection between perceived usefulness and attitudes towards the technology emphasizes the importance of effectively communicating the myriad benefits associated with these translation tools. If students are to fully embrace these tools, they need to perceive tangible benefits in terms of improved translation quality, enhanced effectiveness, and overall increased productivity. By spotlighting how translation technology can be a boon for their translation skills and outcomes, educators and technology providers might facilitate a more favorable disposition and enthusiasm among students.

Within the framework of our research, the perceived usefulness of translation technologies emerged as a significant determinant in shaping students’ attitudes towards their use. This finding is consistent with the theoretical underpinnings of the TAM, which underlines that the perceived usefulness of a technology is significant in influencing users’ attitudes [6]. Our results further validate the premise that when students perceive a clear advantage, such as improved quality, effectiveness, or productivity in their translation work, from utilizing translation technologies, their inclination towards these technologies is enhanced. Additionally, the research brings to light the consequential relationship between attitudes and behavioral intentions within the realm of translation technologies. A favorable disposition towards the application of these technologies is not just a passive sentiment; it actively drives the intent to use them. This link between attitude and behavior is grounded in Ajzen and Fishbein’s [60] conceptualization, which delineates attitude as a pivotal affective evaluation that precedes and informs behavior, which is further exemplified by studies focusing on e-learning systems and ICT adoption [33, 61]. The specific items to measure attitude toward use translation technologies in this study, such as finding translation work more intriguing, the fun aspect of engaging with the technology, or the anticipation of using it in specific job roles, highlight the affective dimension and personal inclination of students towards these technologies. These affective elements not only substantiate the formation of a positive attitude but also reinforce the notion that user experience, beyond mere utility, plays a crucial role in technology acceptance.

## Implications

This research provides implications that are of both theoretical and practical significance. Theoretically, it expanded the TAM by incorporating computer self-efficacy and perceived enjoyment, which offers a useful foundation for further research on translation and language learning technologies acceptance. The extended model provides a broader understanding of users’ intrinsic motivations and individual competencies that shape technology acceptance. The research also responded to calls for applying the TAM in new contexts, to be more specific, the translation technology acceptance among college students.

Practically, the findings suggest several approaches for promoting translation technology acceptance among students. For educators, the findings underscore the necessity to prioritize enhancing students’ computer self-efficacy for its crucial role in shaping students’ perceptions and enjoyment of translation technologies. To foster this, integrating computer literacy and specialized training for translation tools into curricula is essential. Beyond just basic computer skills, this training should equip students with specific skills that make translation technologies more accessible and enjoyable. Furthermore, educators should aim for the integration of translation technologies into standard coursework, making them an integral part of the learning process, rather than optional resources. Establishing interactive mechanisms will also ensure that teaching strategies and tools remain aligned with students’ evolving needs. Moreover, the findings call for a more concerted effort to emphasize the tangible advantages of these technologies, such as improved linguistic abilities and enhanced academic outcomes, and offer solutions to bridge the apparent gap between computer self-efficacy and the perceived use of translation tools.

For technology developers, the research offers clear guidelines on enhancing user acceptance. It’s pivotal to design translation technologies that are not only functional but also enjoyable. Gamifying the experience, with features like rewards, leaderboards, and interactive learning modules, can significantly increase student engagement. At the core of this is the user experience. Given the robust correlation between perceived ease of use and perceived usefulness, developers must prioritize intuitive and user-friendly interfaces. Regular feedback mechanisms are also crucial, because they can shed light on potential areas of improvement, ensuring that translation technologies remain relevant and user-centric. Considering the non-linear relationship between general computer skills and translation tool proficiency, collaboration between developers and academic institutions can yield translation tools tailored more closely to students’ actual needs.

From a policy perspective, the findings suggest a need to focus on holistic and inclusive adoption strategies. Policymakers should be cognizant of the demographic diversity of the student population, especially considering the potential digital divide faced by students from rural backgrounds. Ensuring equitable access and affordability of translation technologies, irrespective of socio-economic backgrounds, should be a policy priority. Additionally, as the study emphasizes the importance of collaboration, policymakers should foster an ecosystem where universities, technology developers, and policymakers work in tandem. Joint efforts can ensure not only the development and application of cutting-edge translation tools but also their seamless integration into higher education. This collaborative approach will be beneficial to addressing the specificities of factors influencing student acceptance of translation technologies.

## Research limitations and future research directions

While this study has provided insights into the factors influencing technology acceptance, it is essential to recognize several limitations that open avenues for future investigation. Firstly, the cross-sectional design may not fully capture the causal relationships among the constructs. Future research could employ longitudinal or experimental designs to better explore these causal dynamics. Secondly, while this study focuses on certain determinants of technology acceptance, it does not account for other possible factors like system quality, motivation, and institutional support. These elements offer fertile ground for future investigation. Thirdly, this study did not examine the potential moderating effects that could influence the relationships within the research model, such as individual differences or contextual factors that may affect the acceptance of translation technology. Future research should consider investigating the role of moderating variables, particularly in the context of translation technology acceptance, to provide a more intricate understanding of these relationships.

## Conclusion

The objective of this study was to investigate the factors that affect the acceptance of translation technologies among college students by proposing and empirically testing an extended TAM. The results revealed that students who are more confident in their computer skills find translation technologies easier and more enjoyable to use. This enjoyment also positively impacts their perception of the technology’s ease of use. The easier the technology is perceived to be, the more useful it is considered. Perceived enjoyment, perceived usefulness, and perceived ease of use all significantly influence the attitude towards using translation technologies, leading to a higher behavioral intention to use them. However, no significant relationship was identified between computer self-efficacy and perceived usefulness, indicating that general computer skills do not inherently lead to perceiving translation technologies as more useful.

The results highlight the importance of fostering computer self-efficacy and integrating features that enhance enjoyment to promote students’ acceptance of translation technologies. This research contributes to the limited body of knowledge on factors influencing translation technology acceptance in educational contexts. The extended TAM also offers a useful foundation for further research on users’ intrinsic drivers of technology adoption. As translation technologies continue proliferating amidst globalization, ongoing research is essential for informing strategies that successfully promote student acceptance and usage of these emerging technologies.

## Supporting information

S1 Dataset (XLSX)
